# Multiple Biomarker Panels for Early Detection of Breast Cancer in Peripheral Blood

**DOI:** 10.1155/2013/781618

**Published:** 2013-11-26

**Authors:** Fan Zhang, Youping Deng, Renee Drabier

**Affiliations:** ^1^Department of Academic and Institutional Resources and Technology, University of North Texas Health Science Center, Fort Worth, 76107, USA; ^2^Department of Forensic and Investigative Genetics, University of North Texas Health Science Center, Fort Worth, 76107, USA; ^3^Department of Internal Medicine Kidston House, Rush University Medical Center, 630 S. Hermitage Avenue, Room 408, Chicago, IL 60612, USA

## Abstract

Detecting breast cancer at early stages can be challenging. Traditional mammography and tissue microarray that have been studied for early breast cancer detection and prediction have many drawbacks. Therefore, there is a need for more reliable diagnostic tools for early detection of breast cancer due to a number of factors and challenges. In the paper, we presented a five-marker panel approach based on SVM for early detection of breast cancer in peripheral blood and show how to use SVM to model the classification and prediction problem of early detection of breast cancer in peripheral blood. We found that the five-marker panel can improve the prediction performance (area under curve) in the testing data set from 0.5826 to 0.7879. Further pathway analysis showed that the top four five-marker panels are associated with signaling, steroid hormones, metabolism, immune system, and hemostasis, which are consistent with previous findings. Our prediction model can serve as a general model for multibiomarker panel discovery in early detection of other cancers.

## 1. Introduction

Traditional methods mostly used for early detection have been regular and periodic self-examination and annual or biannual checkups using mammography and analysis of tissue biopsies. But mammography as a screening tool for early detection has many drawbacks. For example, mammography may not detect small tumors and is often unsatisfactory for younger women, who typically have dense breast tissue. And if a patient does have a suspicious mammogram, a biopsy will probably be done to make the diagnosis. Obtaining tissue biopsies can be difficult for several reasons, including small size of lump, lack of available medical facilities, and patients' reluctance to undergo invasive procedures due to scaring and costs.

In recent years, functional genomics studies using DNA microarrays have been shown effective in differentiating between breast cancer tissues and normal tissues by measuring thousands of differentially expressed genes simultaneously [1–3]. However, early cancer detection and treatment are still challenging. One reason is that obtaining tissue samples for microarray analysis can be still difficult. Another reason is that the signatures of gene expression difference between normal and cancer obtained in different studies are not sufficiently reproducible or informative to be prognostically useful, although they do give valuable insights into the pathogenesis and biology of human tumor metastasis [4]. Moreover, the fact that breast cancer is not a single homogeneous disease but consists of multiple disease status, each arising from a distinct molecular mechanism and having a distinct clinical progression path [5, 6], makes the disease difficult to detect in early stages.

To address these issues, a novel and minimally invasive test that uses easily obtained peripheral blood for breast cancer detection has been developed [7, 8]. For example, Sharma et al. used microarrays and nearest-shrunken-centroid method to analyze the expression pattern of 1,368 genes in peripheral blood cells of 24 women with breast cancer and 32 women with no sign of this disease. The study found that a blood-based gene expression test can be developed to detect breast cancer early in asymptomatic patients [8]. Aarøe et al. collected peripheral blood from 67 breast cancer samples and 63 normal samples, identified a set of 738 differentially expressed probes, and achieved an estimated prediction accuracy of 79.5% with a sensitivity of 80.6% and a specificity of 78.3% [7].

There is a need for more reliable diagnostic tools for early detection of breast cancer in peripheral blood which can achieve high prediction accuracy with as few genes as possible and to reduce the required examination of a large number of genes which increases the dimensionality, computational complexity, and clinical cost of diagnosis [8]. Li estimated that five or six genes rather than 37 or 738 would be sufficient for the early detection of breast cancer, based on colon cancer, leukemia, and breast cancer [8]. Therefore, it is desirable to adopt a “multimarker panel” concept and nontrivial computational methods that can integrate microarray measurement of multiple differential gene expression levels between disease and controls to achieve good performance for clinical genomic development of biomarkers [9].

Support vector machine (SVM) has several unique characteristics as a research tool for prediction in cancer classification applications. One unique characteristic as a specific type of learning algorithm is that it is characterized by the capacity control of the decision function, the use of the kernel functions, and the sparsity of the solution [10]. The second unique characteristic of SVM is that it is established on the unique theory of the structural risk minimization principle to estimate a function by minimizing an upper bound of the generalization error and therefore very resistant to the overfitting problem, eventually achieving a high generalization performance. The third unique characteristic of SVM is that training SVM is equivalent to solving a linearly constrained quadratic programming problem so that the solution of SVM is always unique and globally optimal, unlike neural networks training, which requires nonlinear optimization with the danger of getting stuck at local minima.

For classification and prediction of breast cancer samples, these unique characteristics make SVM appealing as compared with regression-based models or neural network as seen in [11–13]. For example, Liu et al. used SVM to predict the state of breast cancer and found that SVM outperformed K-means cluster and artificial neural network [11]. Henneges et al. applied oscillating search algorithm for feature selection (OSAF) to iteratively improve features for training of Support vector machines (SVM) to better predict breast cancer [12]. They selected 35 out of 51 nucleosides/ribosylated metabolites in the urine of breast cancer women and controls by LC- ITMS coupling for subsequent computational analyses, and they identified 44 pairwise ratios of metabolite features by iterative optimization of SVM. Liu et al. combined genetic algorithm (GA) and all paired (AP) support vector machine (SVM) methods to determine the predictive features for multiclass breast cancer categorization [13].

There has not been any report until this study that applied SVM to the development of multimarker panels for early detection of breast cancer based on peripheral blood. Based on a neural network approach to multibiomarker panel development for LC/MS/MS proteomics profiles we developed [14], we propose for the first time a multimarker panel development solution for early detection of breast cancer in peripheral blood by using a SVM and show how to use SVM to model the classification and prediction problem of early detection of breast cancer in peripheral blood.

## 2. Methods and Materials

### 2.1. Peripheral Blood Data Collection

The peripheral blood data are publicly available through the GEO database with the accession number GSE16443 [7] and were collected with the purpose of determining the potential of gene expression profiling of peripheral blood cells for early detection of blood cancer. It consists of 130 samples with 67 cases and 63 controls. We downloaded the 130 samples which contain 32,879 probes. Then we randomly divided the 130 samples into two groups: group A as a training group and group B as a testing group (Table 1).

### 2.2. Normalization

Per sample normalization was performed to normalize for staining intensity variations among samples. All expression data on a sample were normalized to the 50th percentile of log base 2 of all values on that sample. First, log ratio base 2 transformation was used to transform the data. And then for each probe the median of the log summarized values from all the samples was calculated and subtracted from each of the samples.

### 2.3. Linear Mixed Model

We used the ABI Human Genome Survey Microarray Version 2 to manage and map probe IDs. A full factorial model was used to represent the fixed effect and the random effect which are used to account for group and probe. The expression log ratios value is the final quantity that is fit by a separate analysis of the variance (ANOVA) statistical model for each probe as *y*_*ij*_ using the following:

(1) $$\begin{array}{c} y_{ij}=\mu +T_{i}+S_{j}+\varepsilon_{ij}, \end{array}$$

where *S*_*j*_ ∈ *N*(0, *σ*_1_^2^), *ε*_*ij*_ ∈ *N*(0, *σ* ^2^). Here, *μ* is the mean expression value, *T*_*i*_ is the fixed group effect (caused by the experimental conditions or treatments being evaluated), *S*_*j*_ is the random sample effect (random effects from either individual biological samples or sample preparations), and *ε*_*ij*_ is the within-groups errors. All random effects are assumed independent of each other and independent of the within-groups errors *ε*_*ij*_.

### 2.4. Statistics

Statistical significance was measured by a three-step method. First, we conducted the above linear mixed model to obtain the *P* value of the significance for the group effect. Then we calculated the FDR adjusted *P* value. Last, we calculated the FDR *q* value using the Storey-Tibshirani method [15]. We chose a significance screening filter (*q* < 0.01) to select genes of which we estimated significant differences in the health and breast cancer samples.

### 2.5. Support Vector Machine Analysis

The classification problem of breast cancer can be restricted to consideration of the two-class problem without loss of generality (breast cancer and normal). We used a support vector machine- (SVM-) based methods [16] to develop the classifier for breast cancer from peripheral blood. And then we applied the classifier to predict blind dataset of breast cancer from peripheral blood.

For the use of the support vector machine as an appropriate tool for prediction of the breast cancer, a three-way data split is applied for training, validation, and testing. The training set is used for learning to fit the parameters of the classifier. The validation set is used to tune the parameters of the classifier. And the testing set is used only to assess the performance of the fully-trained classifier. We first randomly split the data into two groups: group A (training group) and group B (testing group), with roughly equal size. Then we use the *k*-fold cross validation on the training group to find the “optimal” parameters for the classifier. Group A is randomly partitioned into *k* subsamples. For each subsample, a cross section of the data is flagged for use as the *validation set*, and a new model is created by training on the remaining data which are the *training set* and not in the subsample. The cross validation process is then repeated *k* times (the folds), with each of the *k* subsamples used exactly once as the validation data. The *k* results from the folds then can be averaged to produce a single estimation. The testing group is used as testing set.

We chose each combination of *N* (*N* = 5 for five-marker panel) out of all the 42 genes differentially expressed in the training group as inputs to the SVM. In order to find the optimal classifier, we presented an optimization method that measures the area under the curve (AUC) for receiver operating characteristics (ROC). In this scheme, we first train SVM for each combination in the training set with 5-fold cross validation. Then, we measured the AUC for each combination in the validation set. Lastly, the optimal combination C* was determined by

(2) $$\begin{array}{c} C^{*}=\underset{C}{\text{argmax}}\text{ AUC}\left({\text{SVM}}_{C},V\right), \end{array}$$

where AUC is the area under the ROC curve of SVM prediction, SVM is the support vector machine, *C* is combination of picking five out of the 42 genes, and *V* is the validation set of training group.

Fivefold cross validation was used to increase the number of estimates and improve the accuracy of the prediction model by avoiding the overfitting. In 5-fold cross validation, the original sample is randomly partitioned into 5 subsamples. Of the 5 subsamples, a single subsample is retained as the validation data for testing the model, and the remaining 4 subsamples are used as training data. The cross validation process is then repeated 5 times, with each of the 5 subsamples used exactly once as the validation data. The 5 results from the folds then can be averaged to produce a single estimation. The advantage of this method over repeated random sub-sampling is that all observations are used for both training and validation, and each observation is used for validation only once.

### 2.6. Pathway Analysis

The Integrated Pathway Analysis Database (IPAD) (http://bioinfo.hsc.unt.edu/ipad/) [17] is used for pathway analysis.

## 3. Results

We downloaded from the Gene Expression Omnibus (accession number GSE16443) [7] the 130 samples with 67 breast cancer and 63 healthy women. After we randomly divided the 130 samples into two groups, group A as training group and group B as testing group (Table 1), we obtained 32 healthy samples and 34 cancer samples in the training set and validation set and 31 healthy samples and 33 cancer samples in the testing set.

We obtained 42 markers in the training group with *q*  value  <0.01. No data from the testing set were utilized in (1) identification of peripheral blood markers or (2) development of the SVM model.

**Figure 1:**
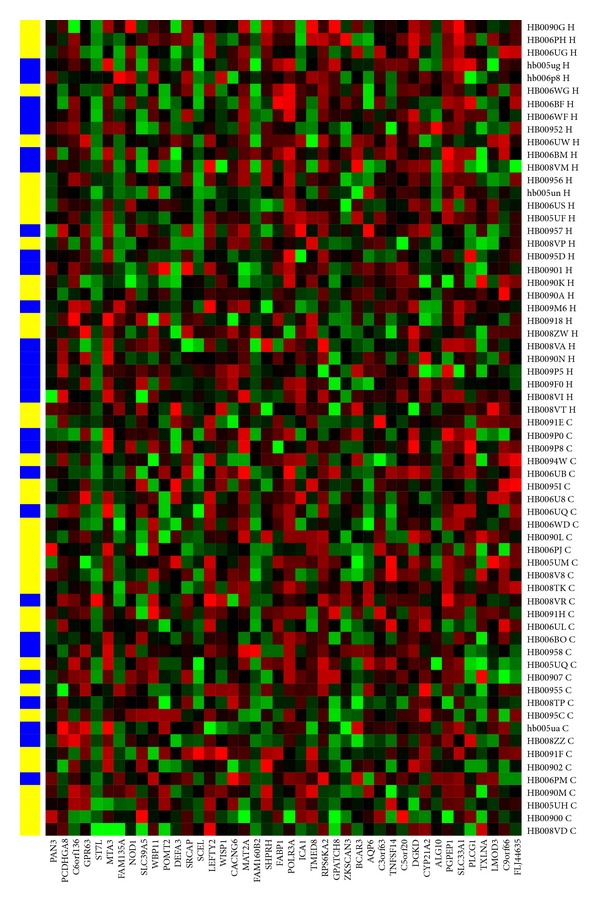
42 biomarkers predicting the healthy and breast cancer samples in testing set. *X*-axis is the 42 biomarkers. *Y*-axis shows the 33 breast cancer and 31 healthy samples (H: healthy, blue; C: cancer, yellow).

An SVM model with 5-fold cross validation was built on all 42 markers in the training group. We obtained a high performance (AUC = 1.0, precision = 94.4%, accuracy = 97.0%, sensitivity = 100.0%, and specificity = 93.8%) for the training group but a low performance (AUC = 0.58, precision = 58.3%, accuracy = 57.8%, sensitivity = 63.6%, and specificity = 51.6%) for the testing group (Figure 1). The result shows that using all markers as a predictor can improve the prediction accuracy only for training group but not for the testing group. Therefore, we constructed an SVM with 5-fold cross validation for each combination of five out of 42 markers and trained with breast cancer cells in peripheral blood derived from 34 women diagnosed with breast cancer and 32 control women in the training group. The three-way data split was applied for training, validation, and testing. The optimal combinations were obtained by our optimization model based on the training set and validation set in the training group.

Training of the SVM was performed using radius basis function (RBF) kernels function and five-fold cross validation. Receiver operating characteristic (ROC) curve and area under curve (AUC) were calculated to help evaluating the predictive performance of the SVM. We choose *N* = 5 for five-marker panel because (1) our pilot study shows that five markers can be enough to achieve a satisfied performance for prediction and classification of cancer [14], (2) previous papers from other labs estimated that five or six genes would be sufficient for the early detection of breast cancer [18], and (3) we expect to achieve high prediction accuracy for breast cancer with as few genes/proteins as possible.

**Figure 2:**
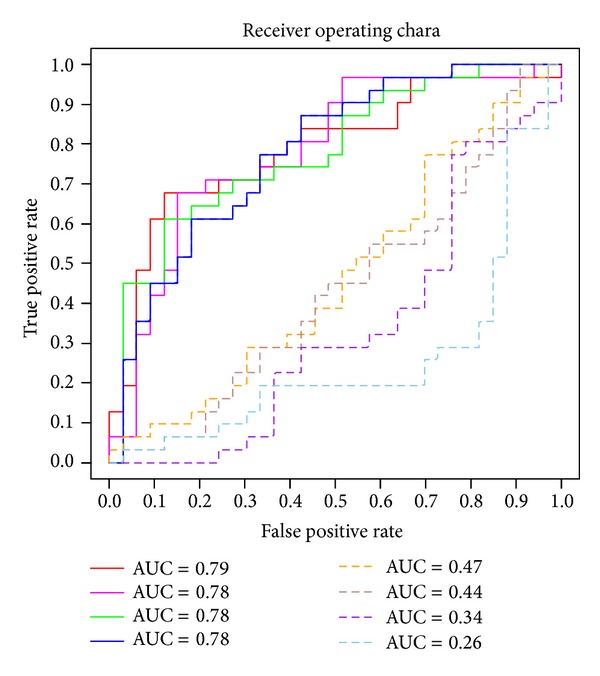
A comparison of best four 5-marker panel ROCs (solid lines) and randomly chosen four (out of 42 candidates) 5-marker ROCs (dotted lines).

In order to validate our prediction method, we compared the ROCs for the best four 5-marker panel predictions determined by our method with the ROCs for four randomly chosen 5-marker panels from 42 candidate biomarkers (Figure 2). As shown in the Figure 2, the top four best predictions determined by our method (solid lines) have better sensitivity-specificity-tradeoff performance than those chosen randomly from 42 candidate biomarkers.

In Table 2, we show the best four five-marker panels identified, using the SVM. Two genes, BCAR3 and LEFTY2, are in common between the best four five-marker panels. Two genes, CACNG6 and DEFA3, are shown three times, and two genes, PCDHGA8 and SCEL, are shown twice.

Pathway analysis shows that the pathways linked with the best four five-marker panels are signaling, steroid hormones, metabolism, immune system, and hemostasis (Table 3), which are consistent with previous findings [7].

The confusion matrix and common performance metrics for both the training group and testing group for the best five-marker panel are shown in the Table 4. Although the final accuracy is 68.75%, it can be considered as an improvement if compared to the original accuracy 58.81%. In addition, the AUC, a comprehensive measurement of sensitivity and specificity, is improved markedly from 0.5826 to 0.7879 (Figure 2 and Table 4).

We further evaluated our multimarker panel prediction performance by comparing our results with prediction performance in previously published findings. Sharma et al. identified a panel of 37 genes that permitted early detection with the classification accuracy of 82% [8], and Aarøe et al. identified a set of 738 differentially expressed probes that achieved an estimated prediction accuracy of 79.5% with a sensitivity of 80.6% and a specificity of 78.3% [7]. Considering that their methods were not applied to independent testing group randomly separated from training group but used *k*-fold cross validation where the original sample was randomly partitioned into *k* subsamples and of the *k* subsamples, a single subsample was retained as the validation data for testing the model, and the remaining *k* − 1 subsamples were used as training data, our prediction performance actually outperformed them. When we applied an SVM and 5-fold cross validation with our best 5-marker panel to the training group of 34 women with breast cancer and 32 healthy women controls, we obtained a higher performance than these previously published findings (precision = 82.86%, accuracy = 83.33%, sensitivity = 85.29%, and specificity = 81.25%, Table 4). We believe that our approach is a significant success, considering that we only used five gene markers in a panel to achieve the prediction performance (AUC = 0.7879, precision = 72.41%, accuracy = 68.75%, sensitivity = 63.64%, and specificity = 74.19%).

## 4. Discussions

In this study, we incorporate the use of a three-way data split in combination with an enumeration method based on SVM. It is a reasonably straightforward application of existing methods and achieves substantially higher prediction performance. In our three-way data split, the testing set is used for the purpose of independent testing only and the validation set is used for tuning the parameters in the SVM training. Splitting the data three ways to get training, validation, and testing sets actually makes our approach very close to real applications. We cannot always select markers based on testing data because in most real applications the testing data are blind or unknown pending for prediction. The prediction performance of the testing set in a three-way data split can actually reflect the outcome in a real application. The best model selected from the training group may not produce the best prediction performance in the testing data due to the inconsistence between the training data and testing data. However, our results show that the selected top models will produce acceptable performance in the testing set, although not best performance.

Although some other researches achieved higher performance, for example, 82% by Sharma et al. [8] and 79.5% by Aarøe et al. [7], our prediction result outperforms theirs if we use training group only (precision = 82.86%, accuracy = 83.33%, sensitivity = 85.29%, and specificity = 81.25%, Table 4) as they did. Our prediction performance which is more close to a real application is actually based on the testing set which is totally blind to the training group (precision = 72.41%, accuracy = 68.75%, sensitivity = 63.64%, and specificity = 74.19%, Table 4).

One limitation of the three-way data split is the sample size. If we split a small size sample into three ways, we would end up with so little data in each set that our analysis would lack any power. If we identify the inconsistence of prediction performance between the validation set and testing set, we can increase the size of training group (training set and validation set) and decrease the size of testing set by simply moving some samples in the testing set to the training group.

Since our approach enumerates all possible combinations of 5 out of *N* markers, there is a limitation for the size of *N* due to current computational capability. In our talon supercomputer, it would take about 1 hour to calculate all combinations of 5 out of 32 markers and about two weeks to finish the computation of picking 5 out of 100 markers. It is acceptable for us to set the maximum of *N* to be 100 because in most cases the top 100 markers can be both specific and sensitive in understanding the treatment, diagnosis, and prognosis of cancer and can be limited by setting a reasonable *P* value threshold.

An ANOVA statistical model is used for identifying differentially expressed genes between cancer and normal samples. For a simple two-group comparison, we would get the identical result if we were to compare the two groups using ANOVA, *t*-test, or SAM. However, ANOVA is a much more flexible and powerful technique that can be applied to much more complex research issues with multiple factors than the other two methods. For example, for the peripheral blood data, we should take into account two factors: (1) the fixed group effect (caused by the experimental conditions or treatments being evaluated) and (2) the random sample effect (random effects from either individual biological samples or sample preparations). In this case, ANOVA method is more efficient than multiple two-group studies analyzed via *t*-test or SAM, because with fewer observations we can gain more information.

In this work, we use the support vector machine (SVM) for classification, which is in general believed to outperform the other classification methods such as the logistic regression (LR) and the artificial neural networks (ANN) [19, 20], because the SVM prediction improves LR and ANN significantly along the specificity axis [21]. However, we understand that for special problems the ANN may still yield reasonable results and that the conclusion that SVM outperforms ANN is in general from a theoretical perspective and in particular for the considered case study [22]. Therefore, we strongly suggest that the tree-way data split method should be carried out for this kind of comparison before we reach any conclusions.

## 5. Conclusions

We developed an integrated computational approach that addressed a challenging multipanel biomarker development problem in the early detection of breast cancer in peripheral blood. The approach that we used combined simple statistical filtering of ANOVA with an optimization model of SVM. The approach automatically learned nonlinear relationships between features and outcomes to generate predictive models, which achieved AUC = 0.7879 performance with a sensitivity of 63.64% and a specificity of 74.19% in the testing data set of 33 women with breast cancer and 31 healthy women controls. The SVM combined with the AUC optimization method is capable of identifying the optimal combination of multimarkers for performance comparable to that of conventional medical decision support systems. We believe that this computational approach works well with early detection of breast cancer in peripheral blood and can provide general guidance for future molecular medicine multimarker panel discovery applications in other diseases. In the future, we will follow up with biological experiments to validate these biomarkers with our collaborators.

## Figures and Tables

**Table 1:** Statistics of samples.

	#health	#cancer	#total
Training group	32	34	66
Testing group	31	33	64

Total	63	67	130

**Table 2:** Best four five-marker panels identified.

Panel	Training group AUC	Testing group AUC
PCDHGA8; LEFTY2; CACNG6; BCAR3; CYP21A2	0.9053	0.7879
PCDHGA8; DEFA3; SCEL; LEFTY2; BCAR3	0.9127	0.7830
DEFA3; SCEL; LEFTY2; CACNG6; BCAR3	0.9154	0.7801
DEFA3; LEFTY2; CACNG6; BCAR3; DGKD	0.8897	0.7801

**Table 3:** Pathway analysis for the best four five-marker panels.

Pathway ID	Pathway name	Molecule
200071	Regulation of CDC42 activity	BCAR3
hsa04260	Cardiac muscle contraction	CACNG6
hsa05412	Arrhythmogenic right ventricular cardiomyopathy (ARVC)	CACNG6
hsa05410	Hypertrophic cardiomyopathy (HCM)	CACNG6
hsa05414	Dilated cardiomyopathy	CACNG6
hsa04010	MAPK signaling pathway	CACNG6
194002	Glucocorticoid biosynthesis	CYP21A2
193993	Mineralocorticoid biosynthesis	CYP21A2
211976	Endogenous sterols	CYP21A2
209943	Steroid hormones	CYP21A2
196071	Metabolism of steroid hormones and vitamins A and D	CYP21A2
211897	Cytochrome P450, arranged by substrate type	CYP21A2
211945	Phase 1, functionalization of compounds	CYP21A2
211859	Biological oxidations	CYP21A2
hsa00140	Steroid hormone biosynthesis	CYP21A2
556833	Metabolism of lipids and lipoproteins	CYP21A2
1430728	Metabolism	CYP21A2
1462054	Alpha-defensins	DEFA3
1461973	Defensins	DEFA3
hsa05202	Transcriptional misregulation in cancer	DEFA3
168249	Innate immune system	DEFA3
168256	Immune system	DEFA3
114508	Effects of PIP2 hydrolysis	DGKD
hsa00561	Glycerolipid metabolism	DGKD
hsa04070	Phosphatidylinositol signaling system	DGKD
hsa00564	Glycerophospholipid metabolism	DGKD
416476	G alpha (q) signalling events	DGKD
388396	GPCR downstream signaling	DGKD
372790	Signaling by GPCR	DGKD
162582	Signal transduction	DGKD
76002	Platelet activation, signaling, and aggregation	DGKD; LEFTY2
109582	Hemostasis	DGKD; LEFTY2
1433617	Regulation of signaling by NODAL	LEFTY2
1181150	Signaling by NODAL	LEFTY2
114608	Platelet degranulation	LEFTY2
76005	Response to elevated platelet cytosolic Ca2+	LEFTY2
hsa04350	TGF-beta signaling pathway	LEFTY2
1266738	Developmental biology	LEFTY2

**Table 4:** Prediction result for the best 5-marker panel.

Predicted	Training group		Testing group	
	Cancer	Normal	Cancer	Normal
Cancer	29	6	21	8
Normal	5	26	12	23
Precision		82.86%		72.41%
Accuracy		83.33%		68.75%
Sensitivity		85.29%		63.64%
Specificity		81.25%		74.19%
